# Tauroursodeoxycholic acid (TUDCA) attenuates pressure overload-induced cardiac remodeling by reducing endoplasmic reticulum stress

**DOI:** 10.1371/journal.pone.0176071

**Published:** 2017-04-20

**Authors:** Shilpa Rani, Pradeep Kumar Sreenivasaiah, Jin Ock Kim, Mi Young Lee, Wan Seok Kang, Yong Sook Kim, Youngkeun Ahn, Woo Jin Park, Chunghee Cho, Do Han Kim

**Affiliations:** 1 School of Life Sciences, Gwangju Institute of Science and Technology (GIST), Gwangju, Korea; 2 Department of Cardiology, Chonnam National University Hospital, Gwangju, Korea; Texas A&M University Health Sciences Center, UNITED STATES

## Abstract

Pressure overload in the heart induces pathological hypertrophy and is associated with cardiac dysfunction. Apoptosis and fibrosis signaling initiated by the endoplasmic reticulum stress (ERS) is known to contribute to these maladaptive effects. The aim of this study was to investigate whether reduction of ERS by a known chemical chaperone, tauroursodeoxycholic acid (TUDCA) can attenuate pressure overload-induced cardiac remodeling in a mouse model of transverse aortic constriction (TAC). Oral administration of TUDCA at a dose of 300 mg/kg body weight (BW) in the TUDCA-TAC group reduced ERS markers (GRP78, p-PERK, and p-eIf2α), compared to the Vehicle (Veh)-TAC group. TUDCA administration, for 4 weeks after TAC significantly reduced cardiac hypertrophy as shown by the reduced heart weight (HW) to BW ratio, and expression of hypertrophic marker genes (*ANF*, *BNP*, and *α-SKA*). Masson's trichrome staining showed that myocardial fibrosis and collagen deposition were also significantly reduced in the TUDCA-TAC group. We also found that TUDCA significantly decreased expression of TGF-β signaling proteins and collagen isoforms. TUDCA administration also reduced cardiac apoptosis and the related proteins in the TUDCA-TAC group. Microarray analysis followed by gene ontology (GO) and pathway analysis demonstrated that extracellular matrix genes responsible for hypertrophy and fibrosis, and mitochondrial genes responsible for apoptosis and fatty acid metabolism were significantly altered in the Veh-TAC group, but the alterations were normalized in the TUDCA-TAC group, suggesting potential of TUDCA in treatment of heart diseases related to pressure-overload.

## Introduction

Cardiac hypertrophy is the myocardial response to various pathological heart diseases, including hypertension, myocardial ischemia, valve disease, and heart failure. Cardiac hypertrophy is characterized by an enhanced protein synthesis, increase in cell size, and heightened organization of the sarcomere [1]. Sustained pathological hypertrophy leads to cardiac remodeling, which is associated with increased interstitial fibrosis and impaired cardiac function.

The endoplasmic reticulum (ER) serves as a major hub for the synthesis and folding of proteins in the cell, hence it plays an important role in maintaining cellular homeostasis. Any perturbation, such as elevated protein synthesis, ischemia, gene mutations, free-radical exposure, or hypoxia can disturb ER homeostasis and cause the pathological accumulation of unfolded/misfolded proteins in the ER, a condition called ER stress (ERS). This subsequently triggers an evolutionarily conserved response called the unfolded protein response (UPR) [2]. The UPR is triggered in cells when ER transmembrane proteins, inositol-requiring enzyme (IRE), protein kinase RNA-like endoplasmic reticulum kinase (PERK), and activating transcription factor 6 (ATF6), detect the accumulation of unfolded proteins. The primary response is the upregulation of ER chaperones such as immunoglobulin heavy-chain-binding protein (BiP)/ glucose regulated protein 78 (GRP78), glucose-regulated protein 94 (GRP94), and calreticulin (CRT), which in turn enhance the ability of the ER to maintain homeostasis of the vital cellular processes by maintaining the intracellular levels of Ca^2+^ and the unfolded proteins [3].

Recent evidence has revealed that ERS plays a pivotal role in the pathogenesis of heart diseases [4, 5]. Apoptotic signals are initiated by the ER, led by induction of CCAAT/enhancer binding protein [C/EBP] homologous protein (CHOP) in heart failure [6]. Cardiac pressure overload is associated with high protein synthesis and Ca^2+^ dysregulation that may lead to sustained ERS, followed by myocardial apoptosis and fibrosis during the transition from cardiac hypertrophy to heart failure [6, 7]. However, relatively less is known about the crosstalk between ERS and pathological remodeling processes. Studies on the signaling molecules such as CaN-MEF2c, JNK, c-Jun, NF-κB, calreticulin and TNFα involved in both ERS pathways and cardiac remodeling have suggested that ERS pathways are highly associated with cardiac remodeling that could cause heart failure [7–10].

Chemical chaperones are small molecules that stabilize misfolded proteins and facilitate their proper folding in a non-selective manner [11]. TUDCA is a hydrophilic bile acid studied extensively in the treatment of ulcerative colitis and primary biliary cirrhosis in preclinical studies [12]. Evidence has shown that TUDCA functions as a chemical chaperone both *in vitro* and *in vivo* [13, 14] although its effects on pressure overload-induced cardiac remodeling have remained to be investigated.

In the present study, we evaluated the effects of TUDCA on pressure overload-induced cardiac remodeling, with respect to myocardial hypertrophy, apoptosis, fibrosis, and associated gene expression. Our results showed that the oral administration of TUDCA attenuated pathological cardiac remodeling by alleviating ERS, suggesting that TUDCA can be a potential therapeutic agent to suppress maladaptive cardiac remodeling.

## Materials and methods

### Ethics statement

All experimental procedures were approved by the Gwangju Institute of Science and Technology Animal Care and Use Committee.

### Transverse aortic constriction (TAC) and administration of TUDCA

Male mice aged 8–10 weeks were used for this study. Mice were anesthetized with 0.3–0.5 ml of 1x Avertin solution (a mixture of 2,2,2-tribromoethanol and tert-amyl alcohol) which was administered via intra-peritoneal injection. The surgical procedure was performed as described previously [15]. The mice were administered TUDCA (Calbiochem, La Jolla, CA, USA) at a dose of 300 mg/kg/day in water by oral gavage (the dosage was determined in a preliminary study, as shown in Figure A in S1 File). Each treatment group consisted of 10–14 animals. TUDCA was administered daily from the day of surgery to both sham- and TAC-operated mice for 1 week or 4 weeks. The control group animals were administered vehicle (water). The animals were monitored daily, and no adverse effects were noticed during the study period. Animals were sacrificed by cervical dislocation after the study (Figure B in S1 File).

The TUDCA dose of 300 mg/kg in mouse corresponds to human equivalent dose (HED) of 24.3 mg/kg. The HED was calculated based on the principle of interspecies allometric drug dose scaling method [16].

### Echocardiographic assessment of left ventricular (LV) function

We performed two-dimensional (2D) guided M-mode echocardiography to evaluate the heart function. Echocardiography was performed after anesthetizing the mice with Avertin solution as described above. A 15-MHz linear array transducer system (iE33 system; Philips Medical Systems, Andover, MA, USA) was used, and the hearts were scanned with the M-mode guided parasternal view.

### Western blot analysis

Western blot analysis was conducted using 50 μg of whole heart lysate, as described previously [15]. The antibodies used are described in S1 File.

### qRT-PCR

Total RNA was extracted from the mouse hearts using TRIzol reagent (Invitrogen Life Technologies, Carlsbad, CA, USA), and cDNAs were produced by reverse-transcribing RNA using the Prime Script RT reagent kit (TaKaRa, Otsu, Japan). qRT-PCR was performed using SYBR Green dye (Kapa Biosystems, Wilmington, MA, USA), and gene expression was normalized to β-actin. The sequences of the specific primers for each of the transcripts are shown in Table A in S1 File.

### Microarray analysis

Total RNA was extracted from the heart samples using TRIzol (Invitrogen) and purified using RNeasy (Qiagen, Valencia, CA, USA). To assess the purity and integrity of the RNA, the OD 260/280 ratio was analyzed using an Agilent 2100 Bioanalyzer (Agilent Technologies, Palo Alto, CA, USA). For performing microarray analysis, labeled RNA (750 ng) was hybridized to a mouse ref-8 expression v.2 bead array for 16–18 h at 58°C, (Illumina Inc., San Diego, CA, USA). Amersham fluorolink streptavidin-Cy3 (GE Healthcare Bio-Sciences, Little Chalfont, UK) was used to analyze the signals. Illumina bead array reader was used to scan the arrays. The GenomeStudio v 2011.1 (Gene Expression Module v1.9.0; Illumina) software was used to extract the raw data.

### Primary cell culture and immunocytochemistry

Neonatal rat ventricular myocytes (NRVMs) were cultured using a neonatal cardiomyocyte isolation system (Worthington Biochemical Corp., Lakewood, NJ, USA), according to the manufacturer’s instructions. The hypertrophic agent was administered as described previously [9]. A brief, description is provided in S1 File.

### Evaluation of apoptosis by TUNEL assay

Heart tissue sections were subjected to TUNEL assay. Apoptosis was examined using the TUNEL assay kit (In Situ Cell Death Detection Kit, TMR red; Roche Applied Science, Penzberg, Germany). Protocol was followed as per manufacturer’s instructions. Nuclear staining was performed with DAPI (Molecular Probes Inc., Eugene, OR, USA). The number of TUNEL-positive nuclei in the heart section was calculated using an IX81 inverted microscope (Olympus) and analyzed using Image J software (NIH Image).

### Histological analysis

Hearts were fixed in 4% paraformaldehyde, paraffin-embedded, and 4–6-μm thick sections were cut using a microtome (RM2135, Leica). These sections were mounted on slides and stained with hematoxylin eosin (H&E, catalog # HHS32 and HT110116, respectively, Sigma-Aldrich) and Masson’s trichrome staining solution (HT15 kit, Sigma-Aldrich), which results in the collagen rich areas appearing blue, and cellular elements appearing red. Images were captured, the percentage of fibrosis was determined from 4–5 images per heart, and ratio of fibrosis to the total area of the cross section was calculated. The fibrotic areas were quantified using Image J software.

### Statistics

The results are expressed as means ± SEM. Comparisons of the group means were made using a one-way or two way ANOVA with a Bonferroni post-hoc test or student’s *t*-test as appropriate and *P* < 0.05 was considered statistically significant.

## Results

### TUDCA alleviated ERS and myocardial hypertrophy in a mouse model of pressure overload

Our previous studies have revealed that ERS proteins are significantly elevated in the heart undergoing pressure overload-induced hypertrophy by transverse aortic constriction (TAC) [9]. To investigate the effect of TUDCA on ERS in the hypertrophied heart, TUDCA, at various dosages, was orally fed to mice after TAC surgery. The hearts were then extracted and ERS markers, such as GRP78 and GRP94, were evaluated (Figure A in S1 File). The results showed that TUDCA dose at 300 mg/kg body weight (BW) significantly decreased the expression levels of GRP78 and GRP94, and decreased heart weight (HW)/BW ratios, compared to the Vehicle (Veh)-TAC group. Therefore, we used this dose throughout the study in all further experiments.

**Fig 1:**
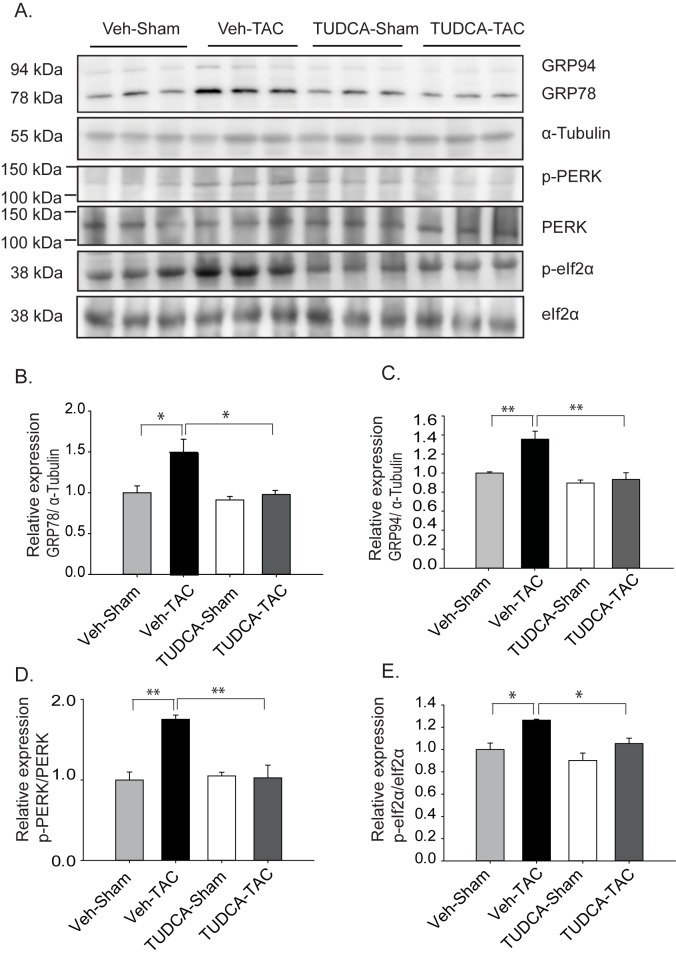
Tauroursodeoxycholic acid (TUDCA) attenuated endoplasmic reticulum stress (ERS) responses in transverse aortic constriction (TAC)-induced hypertrophic hearts. (A) Expression levels of the chaperone proteins GRP78, and GRP94 and the ERS signaling pathway proteins p-PERK and p-eIF2α were significantly increased at 4 weeks after TAC. The western blotting results of whole heart homogenates were obtained from sham- and TAC-operated mice administered vehicle and TUDCA (300 mg kg^−1^ day^−1^). (B–E) Relative expression levels of the ERS signaling pathway proteins GRP78, GRP94, p-PERK and p-eIF2α are shown. All data are shown as mean ± SE (* *P* < 0.05, ** *P* < 0.01, *n* = 3).

Oral administration of TUDCA at 300 mg/kg BW in the TUDCA-TAC group decreased the expression levels of GPR78, GRP94, p-PERK and p-eIF2α at 1 week (Figure C in S1 File) and 4 weeks (Fig 1), compared to the Veh-TAC group, suggesting that TUDCA administration attenuates pressure overload-induced ERS responses in the heart. To study the effect of TUDCA on ERS *in-vitro*, NRVMs were treated with increasing concentrations of TUDCA in the presence of thapsigargin (TG). TG is known to induce ERS by inhibiting the SERCA pump and hence depleting ER Ca^2+^ storage in the ER. In NRVMs, a treatment with 1 μM TG for 24 h significantly induced ERS, as noticed by the increased quantity of chaperone proteins, such as GRP94 and GRP78, and the activation of ERS-related apoptosis signal pathways. Pre-treatment with TUDCA significantly decreased the expression of chaperone proteins, p-eIF2α and CHOP in a dose dependent manner (Figure D in S1 File). Collectively, TUDCA showed a successful reduction of ERS *in-vivo* and *in-vitro*.

**Fig 2:**
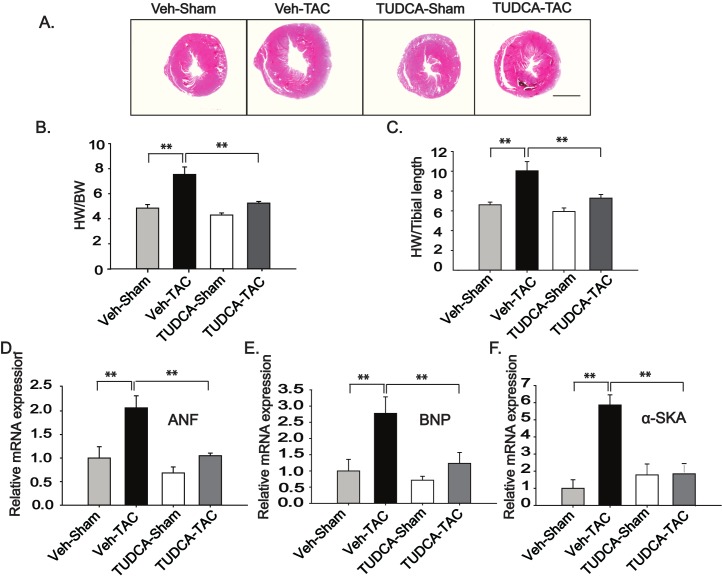
Tauroursodeoxycholic acid (TUDCA) administration reduced hypertrophy in transverse aortic constriction (TAC)-induced hypertrophic hearts. (A) Representative images of whole-heart cross sections obtained by microscopic analysis (hematoxylin-eosin stain) (scale bar = 2 mm). (B, C) Ratios of heart weight (HW)/ body weight (BW) and HW to tibial length as a result of following 4 week TAC and TUDCA administration (*n* = 5–9). (D–F) Transcription levels of *ANF*, *BNP* and *α-SKA* were evaluated by quantitative reverse transcription-polymerase chain reaction using hearts of the sham- and TAC-operated mice after administration of Veh or TUDCA. All data are shown as mean ± SE (* *P* < 0.05, ** *P* < 0.01, *n* = 3).

To evaluate the effect of TUDCA on myocardial hypertrophy, cardiac samples were assessed at 1 week or 4 weeks after TAC surgery by examining the parameters such as heart weight (HW) to BW (HW/BW) ratio and HW to tibia length (HW/TL) ratio. HW/BW and HW/TL were markedly increased in the Veh-TAC group, compared to the Veh-Sham group (Fig 2 and Figure E in S1 File). Daily administration of TUDCA decreased the extent of hypertrophy (Fig 2A–2C). The relative expression levels of the hypertrophy markers, such as atrial natriuretic factor (ANF), brain natriuretic peptide (BNP), and α-skeletal muscle actin (α-SKA), were all significantly increased in the Veh-TAC group, compared to the Veh-Sham group, whereas these markers were decreased in the TUDCA-TAC group (Fig 2D–2F) compared to Veh-TAC group. M-mode echocardiographic examination at 4 weeks after TAC surgery revealed significantly decreased LV fractional shortening (LVFS) (Table B in S1 File) in the Veh-TAC group. However, the TUDCA-TAC group showed a higher LVFS than the Veh-TAC group. In NRVMs, a treatment with phenylephrine (PE) for 24 h significantly increased the cell surface area, whereas the cells treated with PE and TUDCA showed a significantly decreased cell surface area (Figure F in S1 File). Collectively, these findings indicate that the TUDCA treatment attenuated cardiac hypertrophy and improved the cardiac function.

### TUDCA reduced myocardial apoptosis in a mouse model of pressure overload-induced hypertrophy

**Fig 3:**
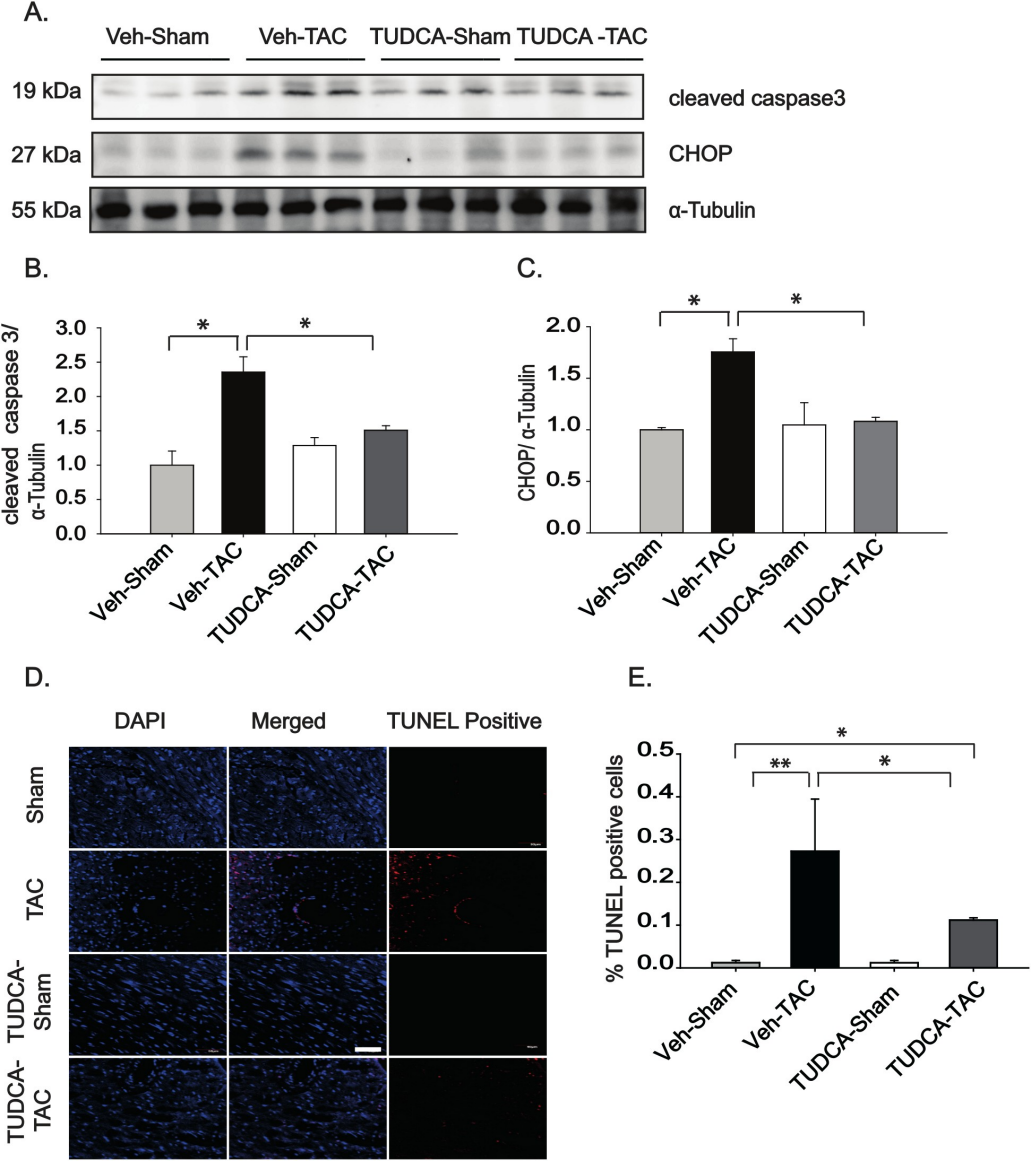
Tauroursodeoxycholic acid (TUDCA) administration reduced the number of apoptotic cells in transverse aortic constriction (TAC)-induced hypertrophic hearts. (A) Protein expression of the pro-apoptotic genes CHOP and cleaved caspase 3 after 4 weeks of TUDCA administration. (B, C) Relative expression of the pro-apoptotic genes CHOP and cleaved caspase 3 (*n* = 3). (D) A TUNEL assay was performed using paraffin-embedded heart tissues (scale bar = 50 μm). (E) Quantitative representation of the percentage of TUNEL-positive cardiomyocytes. Only nuclei that were purplish red were scored. The percentage of TUNEL-positive cells was calculated as the ratio of TUNEL-positive to DAPI-stained nuclei in the section. Three to five fields were selected randomly for each heart section, and quantitated by ImageJ (*n* = 3–6). All data are shown as mean ± SE (* *P* < 0.05, **P < 0.01).

The loss of cardiomyocytes by apoptosis has emerged as an important problem contributing to myocardial remodeling in response to hemodynamic overload [17, 18]. Since prolonged ERS can initiate the apoptotic process through CHOP, we also studied the effect of TUDCA on apoptotic signaling pathway. To evaluate the effect of TUDCA on cardiac apoptosis, heart samples were assessed at 4 weeks after TAC and the concurrent administration of TUDCA (300 mg/kg BW). As shown in Fig 3A–3C, the expressions of cleaved caspase 3 and CHOP were markedly increased in the Veh-TAC group, but TUDCA significantly normalized the expressions in the TUDCA-TAC group. We also examined apoptosis of cardiomyocytes by TUNEL staining. The TUNEL results (expressed as the percentage of positive nuclei per total nuclei) showed low levels of apoptosis in the Veh-Sham group, whereas the level of apoptosis was markedly increased in the Veh-TAC group. In contrast, TUDCA treatment led to a significantly lower rate of TUNEL-positive cells (Fig 3D and 3E).

### TUDCA reduced cardiac fibrosis in a mouse model of pressure overload-induced hypertrophy

**Fig 4:**
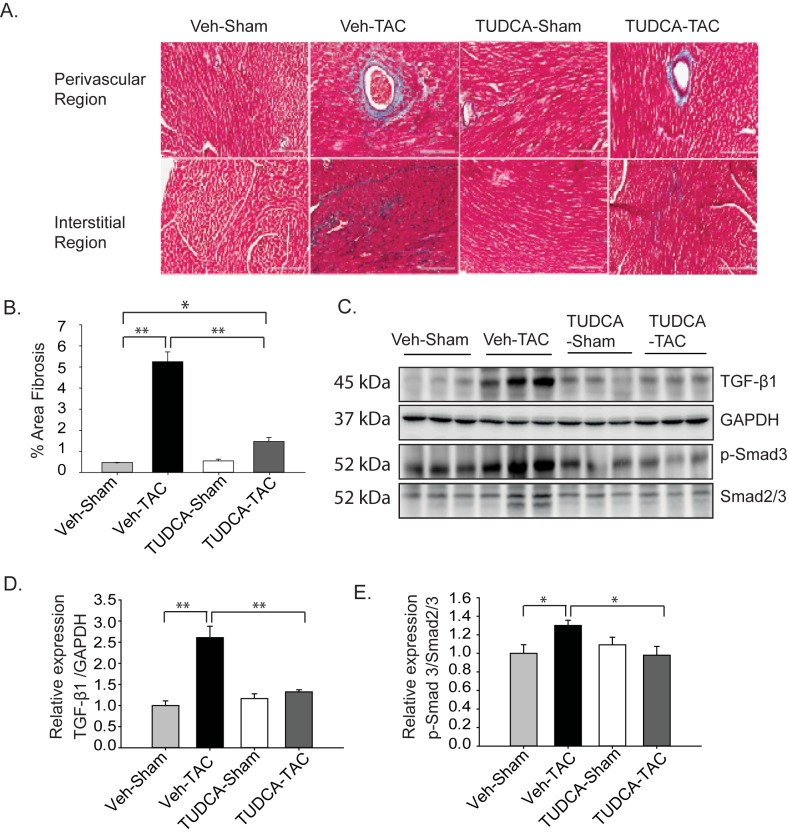
Tauroursodeoxycholic acid (TUDCA) alleviated cardiac fibrosis in transverse aortic constriction (TAC)-induced hypertrophic hearts. (A) Trichrome staining was performed on histological heart sections, as described in **Materials and Methods**. (B) Quantitative representations of the percentage of fibrotic areas are shown. Fibrotic areas were quantified in histological sections using ImageJ. The percentage of fibrosis was determined from 4–5 images per heart focusing on both interstitial and perivascular regions, and calculated as the ratio of fibrosis to the total area of the cross section. (scale bar = 200 μm) (*n* = 3–5). (C) Western blot results of transforming growth factor (TGF)-β1 and Smad proteins in the whole-heart homogenates of TAC mice treated with Veh or TUDCA for 4 weeks are shown. (D, E) Relative expression levels of TGF-β1 and p-Smad proteins are shown. All data are shown as mean ± SE. (* *P* < 0.05, **P < 0.01).

Myocardial fibrosis is another serious symptom that occurs during the transition between myocardial hypertrophy and heart failure. To study the effect of TUDCA on cardiac fibrosis, heart samples were evaluated at 4 weeks after TAC and with concurrent administration of TUDCA (300 mg/kg BW). Cardiac fibrosis is known to be mediated by the activation of the TGF-β receptor, which leads to phosphorylation of Smad2/3. Heart sections were stained with Masson’s trichrome staining to examine the degree of collagen deposition. The results showed significantly decreased collagen deposition in the TUDCA-TAC group, compared to the Veh-TAC group (Fig 4A and 4B). Fig 4C–4E showed that the Veh-TAC group had significantly higher TGF-β and p-Smad3 levels than the Veh-Sham group, but TUDCA administration significantly reduced the protein levels in the TUDCA-TAC group.

**Fig 5:**
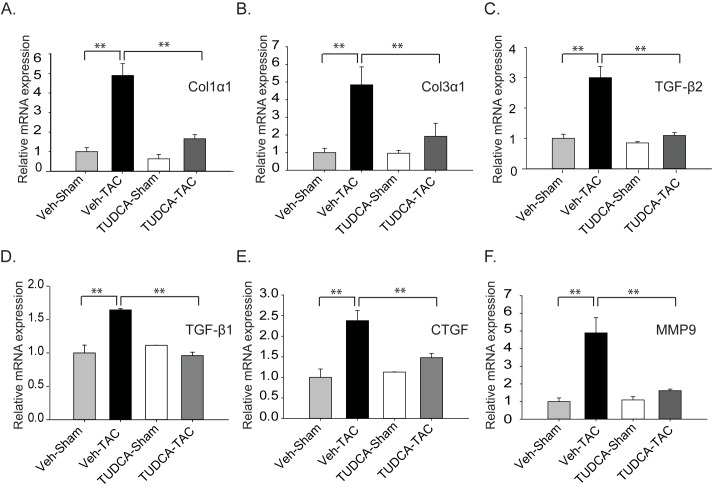
Tauroursodeoxycholic acid (TUDCA) alleviated TAC-induced cardiac fibrosis. (A–E) Transcripts of pro-collagens, TGF-β1, TGF-β2, CTGF, and MMP9 were evaluated by real-time polymerase chain reaction using hearts of the sham- and TAC-operated mice after administration of Veh or TUDCA for 4 weeks. All data are shown as mean ± SE. (* *P* < 0.05, **P < 0.01, *n* = 3).

Relative mRNA expression levels of the fibrosis markers such as collagen 1α1 and collagen 3α1 were significantly lower in the TUDCA-TAC group, than in the Veh-TAC group, as expected (Fig 5). We further investigated the transcription levels of genes involved in cardiac fibrosis such as connective tissue growth factor (CTGF) [19], TGF-β type 1 receptor (TGF β-1), TGF-β type 2 receptor (TGF β-2) [20], and MMP [21–23]. Expression levels of all those genes were significantly lower in the TUDCA-TAC group, than in the Veh-TAC group (Fig 5). Collectively, these results show that the increased collagen deposition observed in the Veh-TAC group could successfully be reduced through inhibition of ERS via treatment with TUDCA.

### TUDCA normalized altered gene expression in a pressure overload-induced hypertrophy

In light of the above data showing substantial evidence that the oral administration of TUDCA in a mouse model of pressure overload-induced hypertrophy can ameliorate the heart malfunctions through normalization of the key hypertrophic symptoms, such as increased cardiac apoptosis and fibrosis, we examined the global cardiac gene expression profiles in all experimental groups by using cDNA microarray technology. The heart samples from TAC followed by 1 week of TUDCA administration was selected for this analysis, because more dynamic changes in gene expression were expected at the onset of cardiac hypertrophy. The microarray data were also validated by qRT-PCR analysis of some selected genes (Figure G in S1 File). Our bioinformatics analysis showed 732 differentially expressed genes (DEGs) at the mRNA level, with more than 1.5-fold change (*P* < 0.05) in the Veh-TAC group compared to the Veh-Sham group. However, the number of DEGs were remarkably less in the TUDCA-TAC group (121), than in the Veh-TAC group (732), as shown in Figure H in S1 File, suggesting that the oral administration of TUDCA normalizes the gene expression changes. Hierarchical clustering of DEGs was performed using the altered DEGs identified both in the Veh-TAC and TUDCA-TAC groups (Figures H and I in S1 File). The heat map shows that a number of DEGs were upregulated (187, red) or downregulated (112, blue) in the Veh-TAC group, but were normalized in the TUDCA-TAC group (Figure I in S1 File).

**Fig 6:**
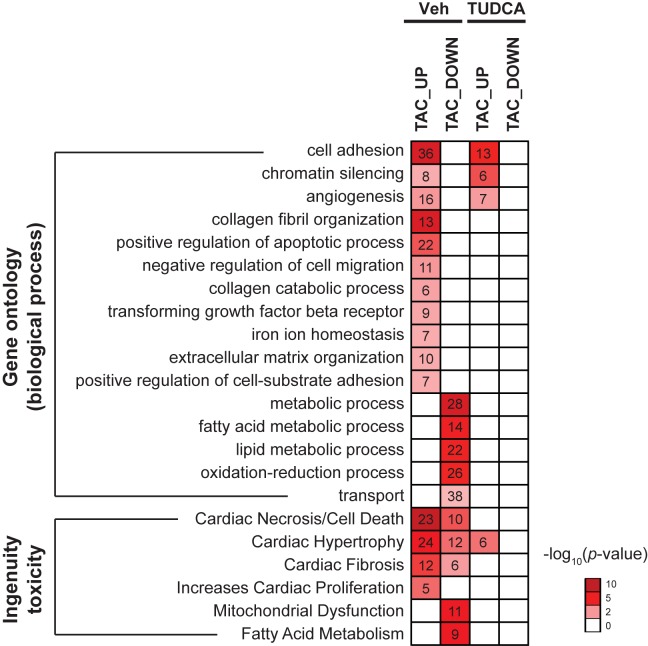
Tauroursodeoxycholic acid (TUDCA) normalized altered gene expression in a pressure overload-induced hypertrophy. The number of significantly reduced DEGs involved in the Gene Ontology (GO) (biological process) or Ingenuity toxicity pathways in the TUDCA-TAC group, compared to the Veh-TAC group. Significantly enriched GO pathways (*P* < 0.05) with at least five DEGs for Veh-TAC or TUDCA-TAC in comparison to control group (Veh-Sham or TUDCA-Sham) are shown. GO analysis was performed using DAVID bioinformatics resources 6.7 (https://david.ncifcrf.gov/)). The Ingenuity toxicity analysis of microarray data was performed by Ingenuity Pathway Analysis (**IPA**) software. Color intensity represents degree of enrichment (-log_10_ [*p*-value]) and the number represents the number of genes.

To investigate the functional effect of TUDCA in the TUDCA-TAC group, we performed gene ontology (GO) analysis of biological functions, as shown in Fig 6. The results showed that TUDCA treatment normalized genes that were overexpressed in the Veh-TAC group, especially those involved in the biological functions such as collagen fibril organization, positive regulation of the apoptotic process, TGF-β receptor, and positive regulation of cell-substrate adhesion. Furthermore, TUDCA also normalized the genes that were downregulated in the Veh-TAC group, which were related to mitochondrial functions such as metabolic process and oxidation-reduction (Fig 6). The Ingenuity toxicity analysis (Fig 6) also showed that the known toxicity items in the heart such as cardiac necrosis/cell death, cardiac fibrosis, increased proliferation, mitochondrial dysfunction were normalized in the TUDCA-TAC group. Collectively, the data showed that TUDCA normalized the DEGs involved in the pathogenesis of the heart in the Veh-TAC group.

## Discussion

Earlier studies have shown that ERS is an important player in the development of pressure overload-induced cardiac hypertrophy [6, 9, 24]. This study examined whether the chemical chaperone TUDCA could ameliorate pressure overload-induced remodeling using an animal model of TAC. The key findings of this study are as follows: (1) TUDCA (oral administration at 300 mg/kg BW) reduced expression of the ERS proteins in the TUDCA-TAC group, compared to Veh-TAC group (Fig 1); (2) TUDCA reduced myocardial hypertrophy in TUDCA-TAC group, compared to the Veh-TAC group (Fig 2); (3) TUDCA ameliorated cardiac apoptosis in the TUDCA-TAC group, compared to the Veh-TAC group (Fig 3); (4) TUDCA attenuated cardiac fibrosis in the TUDCA-TAC group, compared to the Veh-TAC group (Fig 4); (5) TUDCA improved cardiac function as suggested by the increased left ventricular fractional shortening (LVFS) (Table B in S1 File); (6) The microarray data showed that TUDCA administration globally normalized altered gene expressions responsible for the cardiac mal-adaptations by TAC (Fig 6, and Figure I in S1 File).

TUDCA is a nontoxic taurine conjugate form of ursodeoxycholic acid (UDCA) which is an endogenously produced hydrophilic bile acid. In the last decade, TUDCA has been widely studied for its function as a chemical chaperone and its ability to ameliorate ERS and cure various diseases such as biliary fibrosis [25], pulmonary fibrosis [26], retinal degeneration [27], neurodegenerative diseases (e.g. Alzheimer’s, Huntington’s and Parkinson’s diseases) [28] and glucose malfunction [29]. In animal models, TUDCA has shown to have beneficial effects on obesity and high fat-induced myocardial dysfunctions [12, 30] and reduction of apoptosis in mouse model of myocardial infarction [31]. TUDCA also inhibits uremic cardiomyopathy [32]. A recent study showed evidence that TUDCA could ameliorate fibrosis in a mouse model of calreticulin overexpression [10]. To our knowledge, to date, the effects of TUDCA on cardiac remodeling processes by pressure-overload has never been investigated.

In the present study, we found that an oral daily administration of TUDCA at 300 mg/kg BW ameliorated ERS, reduced hypertrophy (Fig 2), and helped restore cardiac contractility as evidenced by the increased fractional shortening in the TUDCA-TAC group (Table B in S1 File). Induction of ER chaperones is a mark of the severity of stress, as seen early in failing human hearts [6]. In this study, TUDCA downregulated ER chaperone proteins, such as GRP78 and GRP94, which are expressed following pressure overload (Fig 1). TUDCA also decreased the phosphorylation of PERK and eIF2α in the TUDCA-TAC group, suggesting that TUDCA administration ameliorates ERS at an early phase, and that subsequently leads to the reduced expression of ER chaperones (Figure J in S1 File). Thus, TUDCA may protect cells in a manner similar to the overexpression of ER resident chaperones, which are known to be cardio-protective [7, 9, 30].

The loss of cardiomyocytes due to enhanced apoptosis, the increased stiffness and lower compliance caused by myocardial fibrosis are two important contributors to the myocardial remodeling that occurs in response to hemodynamic overload [17, 18]. A model of dilated cardiomyopathy showed that ERS-initiated apoptotic signaling is associated with activation of the transcription factor CHOP [33]. Evidence also suggests that CHOP plays an important role in mitochondria-dependent apoptosis in hearts subjected to pressure overload [24]. Similarly, Fig 3 shows upregulation of CHOP and increased apoptotic activity, as shown by increased caspase3-cleavage and TUNEL staining in the Veh-TAC group at 4 weeks after TAC. However, the TUDCA-TAC group showed significantly decreased apoptosis, with lower CHOP and cleaved caspase 3 expression, in accordance with the previously published data, in which TUDCA was shown to decrease apoptosis in a rat infarct heart [31]. Since cardiomyocytes are terminally differentiated cells without the capacity for division, even small quantities of apoptosis, may lead to consistent loss of cardiomyocytes, resulting in serious cardiac dysfunction. Therefore, the present results suggest that the treatment with TUDCA may markedly reduce the cardiac cell death induced by pressure overload.

Myocardial overexpression of TGF-β1 in mice produces ventricular and atrial fibrosis [34–36], whereas its blockade prevented myocardial fibrosis in a rat model of pressure overload [35]. In a recent study, inhibition of UPR by TUDCA in a calreticulin-overexpressing mouse model led to decreased fibrosis and improved cardiac function [10], suggesting a link between ERS and cardiac fibrosis. Here, the TUDCA-TAC group showed attenuated expression of TGF-β and p-Smad3 (Fig 4). The transcription of downstream targets of Smad, collagen 1α1 and collagen 3α1, transcripts were greatly enhanced in the Veh-TAC group, but they were reduced in the TUDCA-TAC group. CTGF, a downstream mediator of the TGF-β pathway, was shown to be induced in hearts of animal models and in cell cultures in response to diverse hypertrophic stimuli [37, 38]. Here, the decreased fibrosis was associated with decreased CTGF expression in the TUDCA-TAC group (Fig 5), similar to a previous study showing decreases in CTGF level by TUDCA treatment in a mouse model of Alzheimer’s disease [28]. The previous studies have shown that enhanced MMP activity is associated with increased fibrosis, whereas attenuated MMP activity is accompanied by decreased fibrosis [21, 39, 40]. In the present study, we observed lower mRNA levels of MMP9 in the TUDCA-TAC group, than in the Veh-TAC group, consistent with the decreased collagen deposition (Fig 4). Pathway analysis using the microarray data also showed decreased expression of ECM-related genes in TUDCA-TAC group.

During pathological cardiac hypertrophy, the heart metabolism undergoes a shift from fatty acid oxidation (FAO) towards glucose utilization. This metabolic switch is coordinated by genes related to FAO and glucose metabolism [41]. ERS is known to be closely associated with mitochondrial damage. Mitochondria play a major role in ER-mediated apoptosis, and TUDCA is known to prevent this apoptosis by blocking Ca^2+^-mediated apoptotic pathways [42]. In the present study, we found that pressure overload-induced cardiac hypertrophy is associated with reduced fatty acid metabolism and mitochondrial gene expression, whereas TUDCA normalized the capacity for fatty acid metabolism and maintained mitochondrial integrity in hypertrophied hearts (Fig 6).

In the initial phase of pathological cardiac hypertrophy, ERS and the following UPR are adaptive processes, while unresolved ERS could eventually lead to maladaptive cardiac remodeling. However, the factors involved in the transition between adaptive and maladaptive phases are still unknown. Hence, we designed the experimental protocol in such a way that the TUDCA administration was started at the onset of cardiac remodeling by TAC.

There was no evidence of nonspecific toxicity by following administration of TUDCA, because we observed no effects on normal growth, weight gain, visceral organ weight, (Table C in S1 File) or physical activity in the study groups.

In conclusion, in the present study, TUDCA effectively suppressed mouse cardiac remodeling by TAC via alleviation of ERS. These results may provide important insights into the cardio-protective effects of TUDCA, which may be clinically relevant for treatment of pressure overload induced cardiac malfunctions.

## Supporting information

S1 File Supplementary materials, figures and tables. (DOCX)

## References

[1] Frey N, Katus HA, Olson EN, Hill JA. Hypertrophy of the heart a new therapeutic target? Circulation. 2004;109:1580–1589. doi: 10.1161/01.CIR.0000120390.68287.BB 15066961 10.1161/01.CIR.0000120390.68287.BB

[2] Yoshida H. ER stress and diseases. FEBS J. 2007;274:630–658. doi: 10.1111/j.1742-4658.2007.05639.x 17288551 10.1111/j.1742-4658.2007.05639.x

[3] Kaufman RJ. Stress signaling from the lumen of the endoplasmic reticulum: coordination of gene transcriptional and translational controls. Genes Dev. 1999;13:1211–1233. 10346810 10.1101/gad.13.10.1211

[4] Groenendyk J, Sreenivasaiah PK, Kim DH, Agellon LB, Michalak M. Biology of endoplasmic reticulum stress in the heart. Circ Res. 2010;107:1185–1197. doi: 10.1161/CIRCRESAHA.110.227033 21071716 10.1161/CIRCRESAHA.110.227033

[5] Minamino T, Komuro I, Kitakaze M. Endoplasmic reticulum stress as a therapeutic target in cardiovascular disease. Circ Res. 2010;107:1071–1082. doi: 10.1161/CIRCRESAHA.110.227819 21030724 10.1161/CIRCRESAHA.110.227819

[6] Okada K, Minamino T, Tsukamoto Y, Liao Y, Tsukamoto O, Takashima S, et al Prolonged endoplasmic reticulum stress in hypertrophic and failing heart after aortic constriction: possible contribution of endoplasmic reticulum stress to cardiac myocyte apoptosis. Circulation. 2004;110:705–712. doi: 10.1161/01.CIR.0000137836.95625.D4 15289376 10.1161/01.CIR.0000137836.95625.D4

[7] Fu HY, Minamino T, Tsukamoto O, Sawada T, Asai M, Kato H, et al Overexpression of endoplasmic reticulum-resident chaperone attenuates cardiomyocyte death induced by proteasome inhibition. Cardiovasc Res. 2008;79:600–610. doi: 10.1093/cvr/cvn128 18508854 10.1093/cvr/cvn128

[8] Hamid T, Guo SZ, Kingery JR, Xiang X, Dawn B, Prabhu SD. Cardiomyocyte NF-κB p65 promotes adverse remodelling, apoptosis, and endoplasmic reticulum stress in heart failure. Cardiovasc Res. 2011;89:129–138. doi: 10.1093/cvr/cvq274 20797985 10.1093/cvr/cvq274 PMC3002872

[9] Park CS, Cha H, Kwon EJ, Sreenivasaiah PK, Kim DH. The chemical chaperone 4-phenylbutyric acid attenuates pressure-overload cardiac hypertrophy by alleviating endoplasmic reticulum stress. Biochem Biophys Res Commun. 2012;421:578–584. doi: 10.1016/j.bbrc.2012.04.048 22525677 10.1016/j.bbrc.2012.04.048

[10] Groenendyk J, Lee D, Jung J, Dyck JR, Lopaschuk GD, Agellon LB, et al Inhibition of the Unfolded Protein Response Mechanism Prevents Cardiac Fibrosis. PLoS One. 2016;11:e0159682 doi: 10.1371/journal.pone.0159682 27441395 10.1371/journal.pone.0159682 PMC4956237

[11] Papp E, Csermely P. Chemical chaperones: mechanisms of action and potential use. Handb Exp Pharmacol. 2006; 172: 405–416. 10.1007/3-540-29717-0_16 16610368

[12] Ceylan-Isik AF, Sreejayan N, Ren J. Endoplasmic reticulum chaperon tauroursodeoxycholic acid alleviates obesity-induced myocardial contractile dysfunction. J Mol Cell Cardiol. 2011;50:107–116. doi: 10.1016/j.yjmcc.2010.10.023 21035453 10.1016/j.yjmcc.2010.10.023 PMC3018539

[13] de Almeida SF, Picarote G, Fleming JV, Carmo-Fonseca M, Azevedo JE, de Sousa M. Chemical chaperones reduce endoplasmic reticulum stress and prevent mutant HFE aggregate formation. J Biol Chem. 2007;282:27905–27912. doi: 10.1074/jbc.M702672200 17626021 10.1074/jbc.M702672200

[14] Chen Y, Liu CP, Xu KF, Mao XD, Lu YB, Fang L, et al Effect of taurine-conjugated ursodeoxycholic acid on endoplasmic reticulum stress and apoptosis induced by advanced glycation end products in cultured mouse podocytes. Am J Nephrol. 2008;28:1014–1022. doi: 10.1159/000148209 18648192 10.1159/000148209

[15] Jeong D, Cha H, Kim E, Kang M, Yang DK, Kim JM, et al PICOT inhibits cardiac hypertrophy and enhances ventricular function and cardiomyocyte contractility. Circ Res. 2006;99:307–314. doi: 10.1161/01.RES.0000234780.06115.2c 16809552 10.1161/01.RES.0000234780.06115.2c

[16] Nair AB, Jacob S. A simple practice guide for dose conversion between animals and human. J Basic Clin Pharma. 2016;7:27–31. 10.4103/0976-0105.177703 PMC4804402 27057123

[17] Katz AM. The cardiomyopathy of overload: an unnatural growth response in the hypertrophied heart. Ann Intern Med. 1994;121:363–371. 8042826 10.7326/0003-4819-121-5-199409010-00009

[18] Narula J, Kolodgie FD, Virmani R. Apoptosis and cardiomyopathy. Curr Opin Cardiol. 2000;15:183–188. 10952426 10.1097/00001573-200005000-00011

[19] Szabó Z, Magga J, Alakoski T, Ulvila J, Piuhola J, Vainio L, et al Connective tissue growth factor inhibition attenuates left ventricular remodeling and dysfunction in pressure overload–induced heart failure. Hypertension. 2014;63:1235–1240. doi: 10.1161/HYPERTENSIONAHA.114.03279 24688123 10.1161/HYPERTENSIONAHA.114.03279

[20] Koitabashi N, Danner T, Zaiman AL, Pinto YM, Rowell J, Mankowski J, et al Pivotal role of cardiomyocyte TGF-β signaling in the murine pathological response to sustained pressure overload. J Clin Invest. 2011;121:2301–2312. doi: 10.1172/JCI44824 21537080 10.1172/JCI44824 PMC3104748

[21] Li YY, Feng YQ, Kadokami T, McTiernan CF, Draviam R, Watkins SC, et al Myocardial extracellular matrix remodeling in transgenic mice overexpressing tumor necrosis factor α can be modulated by anti-tumor necrosis factor α therapy. Proc Natl Acad Sci U S A. 2000;97:12746–12751. doi: 10.1073/pnas.97.23.12746 11070088 10.1073/pnas.97.23.12746 PMC18835

[22] Thomas CV, Coker ML, Zellner JL, Handy JR, Crumbley AJ 3rd, Spinale FG. Increased matrix metalloproteinase activity and selective upregulation in LV myocardium from patients with end-stage dilated cardiomyopathy. Circulation. 1998;97:1708–1715. 9591765 10.1161/01.cir.97.17.1708

[23] Polyakova V, Hein S, Kostin S, Ziegelhoeffer T, Schaper J. Matrix metalloproteinases and their tissue inhibitors in pressure-overloaded human myocardium during heart failure progression. J Am Coll Cardiol. 2004;44:1609–1618. doi: 10.1016/j.jacc.2004.07.023 15489093 10.1016/j.jacc.2004.07.023

[24] Fu HY, Okada K, Liao Y, Tsukamoto O, Isomura T, Asai M, et al Ablation of C/EBP homologous protein attenuates endoplasmic reticulum-mediated apoptosis and cardiac dysfunction induced by pressure overload. Circulation. 2010;122:361–369 doi: 10.1161/CIRCULATIONAHA.109.917914 20625112 10.1161/CIRCULATIONAHA.109.917914

[25] Ben Mosbah I, Alfany-Fernández I, Martel C, Zaouali MA, Bintanel-Morcillo M, Rimola A, et al Endoplasmic reticulum stress inhibition protects steatotic and non-steatotic livers in partial hepatectomy under ischemia–reperfusion. Cell Death Dis. 2010;1:e52 doi: 10.1038/cddis.2010.29 21364657 10.1038/cddis.2010.29 PMC3032561

[26] Baek HA, Kim DS, Park HS, Jang KY, Kang MJ, Lee DG, et al Involvement of endoplasmic reticulum stress in myofibroblastic differentiation of lung fibroblasts. Am J Respir Cell Mol Biol. 2012;46:731–739. doi: 10.1165/rcmb.2011-0121OC 21852685 10.1165/rcmb.2011-0121OC

[27] Drack AV, Dumitrescu AV, Bhattarai S, Gratie D, Stone EM, Mullins R, et al TUDCA slows retinal degeneration in two different mouse models of retinitis pigmentosa and prevents obesity in Bardet-Biedl syndrome type 1 mice. Invest Ophthalmol Vis Sci. 2012;53:100–106. doi: 10.1167/iovs.11-8544 22110077 10.1167/iovs.11-8544 PMC3292352

[28] Lo AC, Callaerts-Vegh Z, Nunes AF, Rodrigues CM, D'Hooge R. Tauroursodeoxycholic acid (TUDCA) supplementation prevents cognitive impairment and amyloid deposition in APP/PS1 mice. Neurobiol Dis. 2013;50:21–29. doi: 10.1016/j.nbd.2012.09.003 22974733 10.1016/j.nbd.2012.09.003

[29] Vettorazzia JF, Ribeiro RA, Borck PC, Branco RC, Soriano S, Merino B, et al The bile acid TUDCA increases glucose-induced insulin secretion via the cAMP/PKA pathway in pancreatic beta cells. Metabolism. 2016;65:54–63. 10.1016/j.metabol.2015.10.021 26892516

[30] Turdi S, Hu N, Ren J. Tauroursodeoxycholic acid mitigates high fat diet-induced cardiomyocyte contractile and intracellular Ca 2+ anomalies. PLoS One. 2013;8:e63615 doi: 10.1371/journal.pone.0063615 23667647 10.1371/journal.pone.0063615 PMC3647067

[31] Rivard AL, Steer CJ, Kren BT, Rodrigues CM, Castro RE, Bianco RW, et al Administration of tauroursodeoxycholic acid (TUDCA) reduces apoptosis following myocardial infarction in rat. Am J Chin Med. 2007;35:279–295. doi: 10.1142/S0192415X07004813 17436368 10.1142/S0192415X07004813

[32] Ding W, Wang B, Zhang M, Gu Y. Involvement of Endoplasmic Reticulum Stress in Uremic Cardiomyopathy: Protective Effects of Tauroursodeoxycholic Acid. Cell Physiol Biochem. 2016;38:141–152. doi: 10.1159/000438616 26765262 10.1159/000438616

[33] Hamada H, Suzuki M, Yuasa S, Mimura N, Shinozuka N, Takada Y, et al Dilated cardiomyopathy caused by aberrant endoplasmic reticulum quality control in mutant KDEL receptor transgenic mice. Mol Cell Biol. 2004;24:8007–8017. doi: 10.1128/MCB.24.18.8007-8017.2004 15340063 10.1128/MCB.24.18.8007-8017.2004 PMC515036

[34] Rosenkranz S, Flesch M, Amann K, Haeuseler C, Kilter H, Seeland U, et al Alterations of β-adrenergic signaling and cardiac hypertrophy in transgenic mice overexpressing TGF-β1. Am J Physiol Heart Circ Physiol. 2002;283:H1253–H1262. doi: 10.1152/ajpheart.00578.2001 12181157 10.1152/ajpheart.00578.2001

[35] Kuwahara F, Kai H, Tokuda K, Kai M, Takeshita A, Egashira K, et al Transforming growth factor-β function blocking prevents myocardial fibrosis and diastolic dysfunction in pressure-overloaded rats. Circulation. 2002;106:130–135. 12093782 10.1161/01.cir.0000020689.12472.e0

[36] Nakajima H, Nakajima HO, Salcher O, Dittiè AS, Dembowsky K, Jing S, et al Atrial but not ventricular fibrosis in mice expressing a mutant transforming growth factor-β1 transgene in the heart. Circ Res. 2000;86:571–579. 10720419 10.1161/01.res.86.5.571

[37] Finckenberg P, Inkinen K, Ahonen J, Merasto S, Louhelainen M, Vapaatalo H, et al Angiotensin II induces connective tissue growth factor gene expression via calcineurin-dependent pathways. Am J Pathol. 2003;163:355–366. doi: 10.1016/S0002-9440(10)63659-0 12819040 10.1016/S0002-9440(10)63659-0 PMC1868168

[38] He Z, Way KJ, Arikawa E, Chou E, Opland DM, Clermont A, et al Differential regulation of angiotensin II-induced expression of connective tissue growth factor by protein kinase C isoforms in the myocardium. J Biol Chem. 2005;280:15719–15726. doi: 10.1074/jbc.M413493200 15699040 10.1074/jbc.M413493200

[39] Dixon IM, Ju H, Reid NL, Scammell-La Fleur T, Werner JP, Jasmin G. Cardiac collagen remodeling in the cardiomyopathic Syrian hamster and the effect of losartan. J Mol Cell Cardiol. 1997;29:1837–1850. doi: 10.1006/jmcc.1997.0420 9236138 10.1006/jmcc.1997.0420

[40] Cowan KN, Jones PL, Rabinovitch M. Regression of hypertrophied rat pulmonary arteries in organ culture is associated with suppression of proteolytic activity, inhibition of tenascin-C, and smooth muscle cell apoptosis. Circ Res. 1999;84:1223–1233. 10347097 10.1161/01.res.84.10.1223

[41] Zhou LY, Liu JP, Wang K, Gao J, Ding SL, Jiao JQ, et al Mitochondrial function in cardiac hypertrophy. Int J Cardiol. 2013;167:1118–1125. doi: 10.1016/j.ijcard.2012.09.082 23044430 10.1016/j.ijcard.2012.09.082

[42] Xie Q, Khaoustov VI, Chung CC, Sohn J, Krishnan B, Lewis DE, et al Effect of tauroursodeoxycholic acid on endoplasmic reticulum stress–induced caspase‐12 activation. Hepatology. 2002;36:592–601. doi: 10.1053/jhep.2002.35441 12198651 10.1053/jhep.2002.35441

