# Supplementary material for: Tauroursodeoxycholic acid (TUDCA) attenuates pressure overload-induced cardiac remodeling by reducing endoplasmic reticulum stress
Source: PLoS One. 2017 Apr 20;12(4):e0176071. doi: 10.1371/journal.pone.0176071 (PMC5398705; doi:10.1371/journal.pone.0176071)
Supplement: S1 File — (DOCX) [file pone.0176071.s001.docx]

**Supporting Information**

**Material and Methods**

**Transverse Aortic Constriction (TAC) and Administration of TUDCA**

This study was performed using male mice of 8-10 weeks of age (25–27 g). Briefly the animals were anesthetized and were ventilated with a tidal volume of 0.1 ml and a respiratory rate of 120 breaths per minute (Harvard Apparatus, Cambridge, MA, USA). A longitudinal incision was made on the sternum to visualize the aortic arch. A ligation was made between the innominate and left common carotid arteries with an overlaid 27-gauge needle, and then the needle was removed leaving a constriction. Animals undergoing the sham operation (the same procedure without constriction) were considered the control group.

**Western Blot Analysis**

Whole heart lysates were obtained by solubilizing cardiac tissue with RIPA buffer (50 mM Tris–HCl (pH 7.4), 150 mM NaCl, 1% NP-40, 0.5% Sodium deoxycholate and protease inhibitor cocktail). Protein concentrations were evaluated using the BCA protein assay kit (Pierce, Rockford, IL, USA). A total of 50 μg of heart tissue lysate was separated by SDS-PAGE and transferred to a polyvinylidene difluoride membrane (PVDF, EMD Millipore Corp. Billerica, MA, USA). The membranes were blocked with 5% skim milk in TBST (pH 7.4) at room temperature for 1 h. The membranes were then incubated with antibodies against p-PERK (Santa Cruz), PERK (Cell Signaling), cleaved caspase3 (Cell Signaling), Smad2/3 (Cell Signaling), p-Smad3 (Cell Signaling), TGF-β (Cell Signaling), p-eIF2α (ser51) (Cell Signaling), eIF2α (Santa Cruz), KDEL (Abcam), CHOP (Cell Signaling) and α-Tubulin (Santa Cruz). The blots were exposed to primary antibody overnight at 4 ̊C. The membranes were incubated with a secondary antibody conjugated to horseradish peroxidase (HRP) (Jackson ImmunoResearch, West Grove, PA, USA) at room temperature for 1 h. The intensity of the signal was detected using an ImageQuant LAS 4000 mini (GE Healthcare Bio-Sciences Chicago, IL, USA) and a SuperSignal West Pico Chemiluminescence Kit (Thermo Fisher Scientific). The protein band intensities were analyzed by ImageJ software (NIH).

**Primary Cell Culture and Immunocytochemistry**

Neonatal rat ventricular myocytes (NRVMs) were isolated using neonatal cardiomyocyte isolation system (Worthington), according to the manufacturer’s instructions. Cardiomyocytes were cultured in Dulbecco's modified Eagle's medium (DMEM) supplemented with 10% FBS (GIBCO, Life Technologies, USA).

Cardiomyocytes were cultured in serum-free medium for at least 24 h and then treated with 100 µM phenylephrine (PE) with or without 300 µM TUDCA for 24 h. The cells were then fixed with 4% paraformaldehyde for 10 min, permeabilized with 0.5% Triton X-100 in PBS for 5 min, and blocked by incubation with 5% bovine serum albumin (BSA) for 1 h at room temperature. The cells were incubated with anti-α actinin antibody (Sigma-Aldrich) and further incubated with Alexa 488-conjugated anti-mouse immunoglobulin (Jackson ImmunoResearch). Immunofluorescence was analyzed using an LSM-700 confocal microscope (Carl Zeiss Inc., Oberkochen, Germany).

**Table A. List of primer sequences used for RT-PCR in the study**

| **Gene Name** | **Forward** | **Reverse** |
| --- | --- | --- |
| ANF | 5′ACCTGCTAGACCACCTGGAGG3′ | 5′GCTGTTATCTTCGGTACCGG3′ |
| BNP | 5’AGGGAGAACACGGCATCATT3’ | 5’GACAGCACCTTCAGGAGAT3’ |
| Collagen1α1 | 5’CGAAGGCAACAGTCGCTTCA3’ | 5’GGTCTTGGTGGTTTTGTATTCGAT3’ |
| Collagen3α1 | 5’GTCCACGAGGTGACAAAGGT3’ | 5’GATGCCCACTTGTTCCATCT3’ |
| β-actin | 5’CACACTGTGCCCATCTACGA3’ | 5’CCATCTCCTGCTCGAAGTCT3’ |
| MMP2 | 5’CCCCGATCTACACCTACACCAAGAAC3’ | 5’CATTCCAGGAGTCTGCGATGAGC3’ |
| CTGF | 5’GGGCCTCTTCTGCGATTTC3’ | 5’ATCCAGGCAAGTGCATTGGTA3’ |
| TGF-β2 | 5’AGCGCTACATCGATAGCAAG3’ | 5’TCCTGTCTTTGTGGTGAAGC3’ |
| MMP9 | 5’TAGTGAGAGACTCTACACGG3’ | 5’CCACTTCTTGTCAGTGTCGA3’ |
| Nox4 | 5’GAAGATTTGCCTGGAAGAACC3’ | 5’AGGTTTGTTGCTCCTGATGC3’ |
| α-ska | 5’TCAGGCGGTGCTGTCTCTCT3’ | 5’TCCCCAGAATCCAACACGAT3’ |
| TGF-β1 | 5’CAACAATTCCTGGCGTTACCTTGG3’ | 5’GAAAGCCCTGTATTCCGTCTCCTT3’ |

**Table B. Echocardiographic data derived from the different experimental animal groups**

|  | Veh-Sham  (n = 5) | Veh-TAC  (n = 7) | TUDCA-Sham  (n = 4) | TUDCA-TAC  (n = 9) |
| --- | --- | --- | --- | --- |
| FS (%) | 39.59 ± 2.18 | 28.60 ± 1.19** | 37.76 ± 1.91 | 35.74 ± 0.99^##^ |
| EF (%) | 76.55 ± 2.43 | 61.94 ± 1.83** | 74.54 ± 2.34 | 72.04 ± 1.37^##^ |
| IVSs (cm) | 0.106 ± 0.006 | 0.094 ± 0.002 | 0.095 ± 0.002 | 0.107 ± 0.002 |
| LVIDd (cm) | 0.316 ± 0.011 | 0.381 ± 0.017* | 0.325 ± 0.022 | 0.322 ± 0.015^#^ |
| LVIDs (cm) | 0.192 ± 0.123 | 0.272 ± 0.014** | 0.202 ± 0.018 | 0.206 ± 0.012^##^ |
| IVSd (cm) | 0.072 ± 0.005 | 0.071 ± 0.001 | 0.067 ± 0.004 | 0.081 ± 0.002 |
| Heart rate (bpm) | 219.0 ± 9.64 | 195.33 ± 9.29 | 222.5 ± 7.84 | 197.37 ± 5.96 |
| Rectal temperature (◦C) | 32.42 ± 0.52 | 31.94 ± 0.38 | 31.95 ± 0.66 | 32.14 ± 0.35 |

Echocardiographic parameters obtained from the different animal groups (Veh-Sham, Veh-TAC, TUDCA-Sham and TUDCA-TAC) after 4 weeks of TUDCA administration. FS, fractional shortening; EF, ejection fraction; IVSd, interventricular septal thickness at diastole; LVIDd, left ventricular internal dimension diastole; LVIDs, left ventricular internal dimension systole; IVSs, interventricular septal thickness at systole. All data are shown as means ± SE (* *P* < 0.05, ** *P* < 0.01 vs. Veh-Sham, # *P* < 0.05, ## *P* < 0.01 vs. Veh-TAC).

**Table C. Biometrics for the experimental animal groups with or without oral treatment of TUDCA for 4 weeks at the dose of 300 mg/kg BW**

|  | Veh-Sham  (n = 5) | Veh-TAC  (n = 6) | TUDCA-Sham  (n = 7) | TUDCA-TAC  (n = 8) |
| --- | --- | --- | --- | --- |
| Body Weight (g) | 28.534 ± 0.990 | 29.633 ± 0.935 | 28.128 ± 0.608 | 27.812 ± 0.757 |
| Heart Weight (g) | 0.137 ± 0.005 | 0.222 ± 0.15** | 0.121 ± 0.006 | 0.146 ± 0.007^##^ |
| HW/BW (mg/g) | 4.847 ± 0.283 | 7.53 ± 0.558** | 4.295 ± 0.171 | 5.24 ± 0.137^##^ |
| Liver Weight (g) | 1.501 ± 0.081 | 1.533 ± 0.089 | 1.319 ± 0.067 | 1.303 ± 0.060 |
| LW/BW (mg/g) | 52.571 ± 1.829 | 52.094 ± 3.943 | 46.805 ± 1.711 | 46.751 ± 1.448 |
| Kidney Weight (g) | 0.42 ± 0.029 | 0.403 ± 0.012 | 0.361 ± 0.027 | 0.344 ± 0.012 |
| KW/BW (mg/kg) | 14.642 ± 0.587 | 13.724 ± 0.864 | 12.885 ± 0.984 | 12.42 ± 0.469 |
| lung weight (g) | 0.194 ± 0.005 | 0.234 ± 0.023 | 0.191 ± 0.006 | 0.191 ± 0.008 |
| Lung weight/BW (mg/g) | 6.840 ± 0.262 | 7.985 ± 1.836 | 6.795 ± 0.219 | 6.893 ± 0.430 |

All data are shown as mean ± SE (* *P* < 0.05, ** *P* < 0.01 vs. Veh-Sham, # *P* < 0.05, ## *P* < 0.01 vs. Veh-TAC).


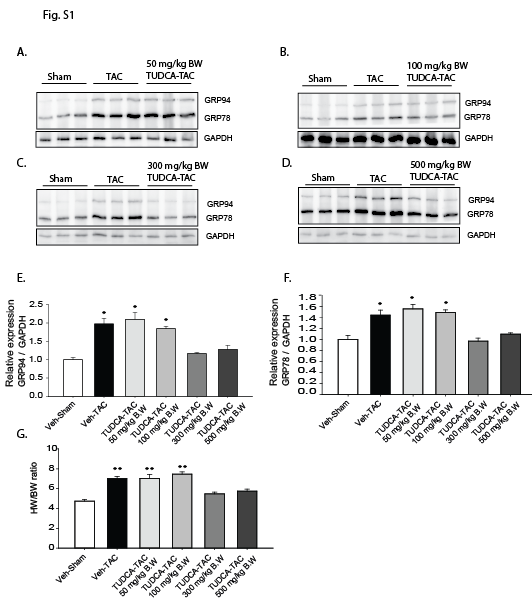


**Figure A. Preliminary evaluation for dose-dependence of tauroursodeoxycholic acid (TUDCA) administration on ERS markers.** (A-F) TUDCA was orally fed at the onset of TAC using different doses for 1 week. The hearts were then evaluated for ERS marker GRP78 and GRP 94 (*n* = 3). (G) HW/BW ratio of the different experimental animal groups (*n* = 4 ­-7). All data are shown as mean ± SE (* P < 0.05, **P < 0.01 vs Veh Sham).


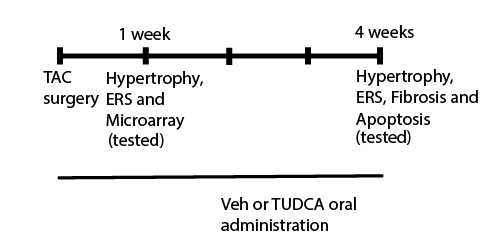


**Figure B. Timelines of the experimental protocols which were conducted.**


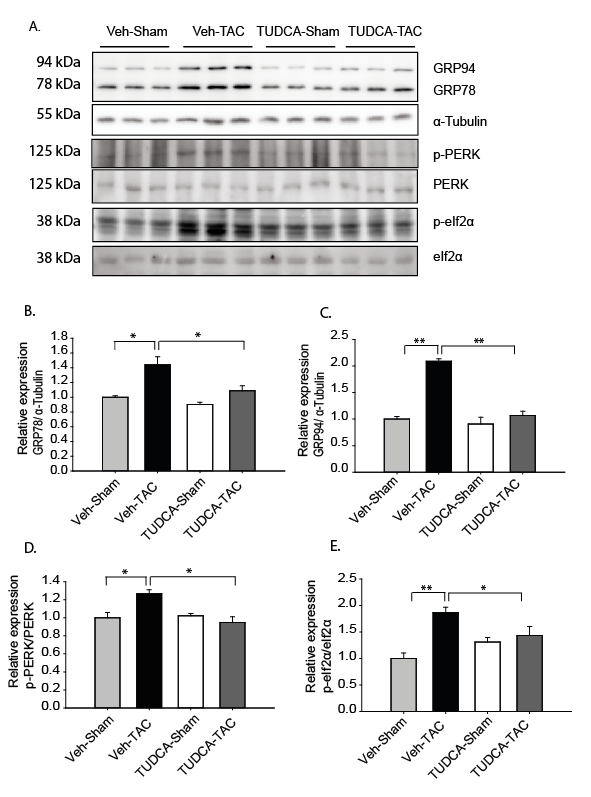


**Figure C. Tauroursodeoxycholic acid (TUDCA) attenuated endoplasmic reticulum stress (ERS) responses in transverse aortic constriction (TAC)-induced hypertrophic hearts (1 week).** (A) Expression levels of the chaperone proteins GRP78 and GRP94 and ERS signaling pathway proteins p-PERK and p-eIF2α at 1 week after TAC. (B-E) Relative expression levels of the GRP78, GRP94, p-PERK and p-eIF2α. All data are shown as mean ± SE (* P < 0.05, **P < 0.01)


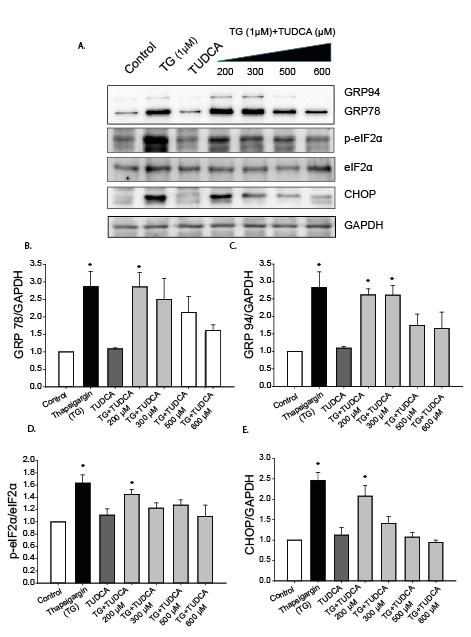


**Figure D. TUDCA protects cardiomyocytes against ER stress** (A) Expression of ERS signaling molecules during thapsigargin (TG) (1µM) induced ERS in NRVM with increasing concentration of TUDCA after 24hrs (* P < 0.05 Vs. control)


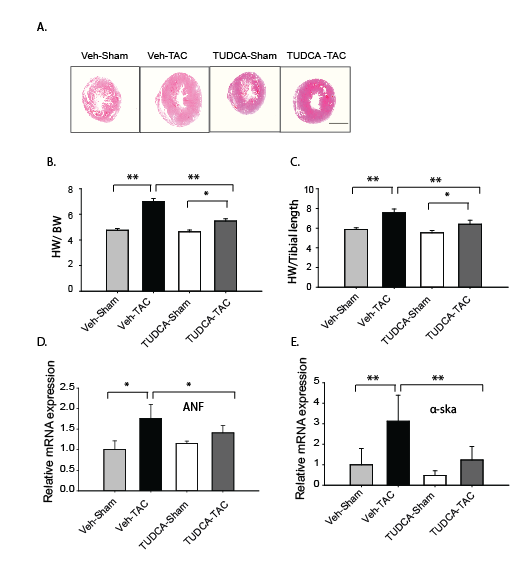


**Figure E. Tauroursodeoxycholic acid (TUDCA) administration reduced hypertrophy in transverse aortic constriction (TAC)-induced hypertrophic hearts (1 week).** (A) Representative images of whole-heart cross sections obtained by microscopic analysis (hematoxylin-eosin stain) (scale bar = 2mm). (B, C) Ratios of heart weight (HW)/ body weight (BW) and HW to tibial length as a result of following 1 week TAC and TUDCA administration. (*n* = 4 - 7) (D–E) Transcription levels of *ANF* and *α-SKA* were evaluated by quantitative reverse transcription-polymerase chain reaction using hearts of the sham- and TAC-operated mice after administration of Veh or TUDCA (*n* = 3). All data are shown as mean ± SE (* P < 0.05, **P < 0.01)


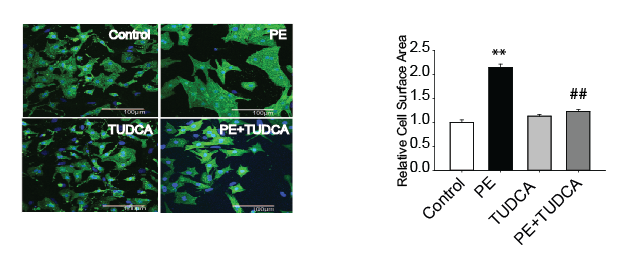


**Figure F: Immunofluorescence image showing the cell sizes of neonatal rat ventricular myocytes** (NRVMs) treated with 100 μM phenylephrine (PE) in the presence or absence of 300 μM TUDCA. All data are shown as mean ± SE (** P < 0.01, vs. Control, ## P < 0.01, vs. PE). Fold increase of cell surface area in the immunofluorescence results. (*n* = 100 cells for each group) (scale bar = 100 µm).


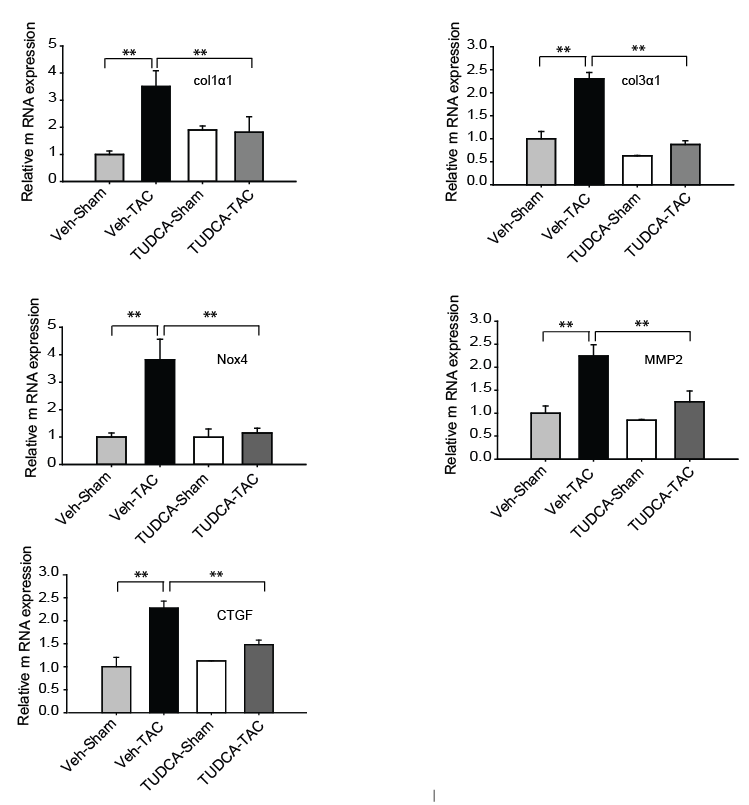
**Figure G. Confirmation of the microarray data with transcript levels of genes evaluated by qRT-PCR after 1 week TAC (* P < 0.05, ** P < 0.01, n = 3).**


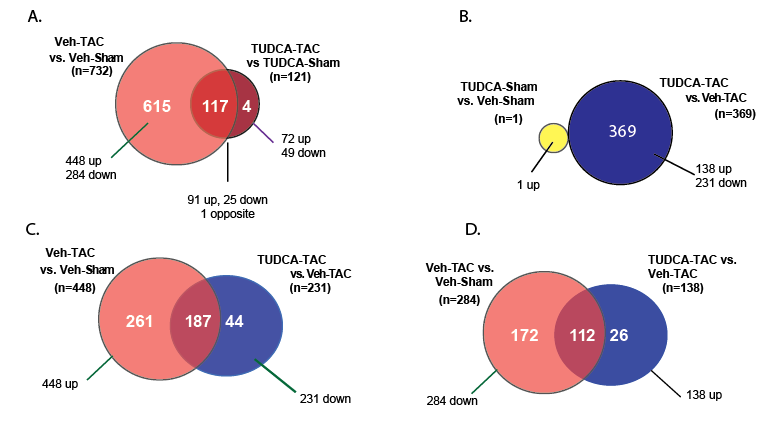


**Figure H. (A) Venn diagrams showing the number of DEGs in Veh-TAC vs. Veh Sham (n = 732) and TUDCA-TAC vs. TUDCA-Sham (n = 121) that were either unique or overlapping in each indicated group.** (B) Venn diagram showing the number of DEGs in TUDCA-Sham vs. Veh-Sham (n = 1) and TUDCA-TAC vs. Veh-TAC (n = 369). (C) Venn diagram showing the number of DEGs upregulated in Veh-TAC (n = 448), but normalized in TUDCA-TAC (n = 187), and (D) Venn diagram showing the number of DEGs down regulated in Veh-TAC (n = 284), but normalized in TUDCA-TAC (n = 112).


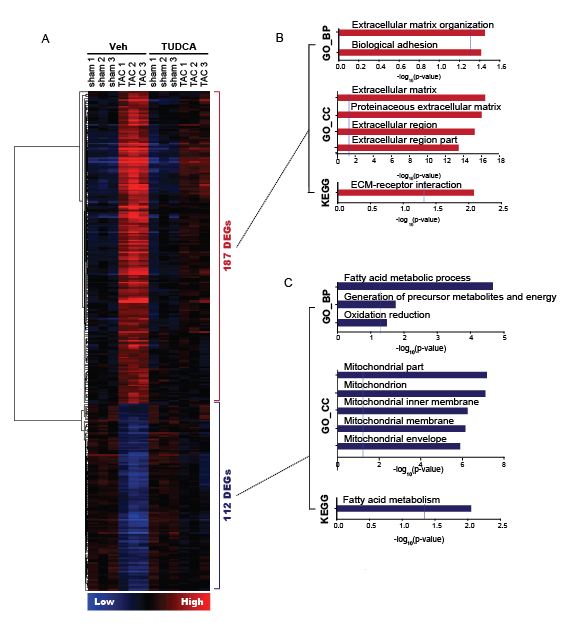


**Figure I. (A) Hierarchical clustering of DEGs significantly altered in the Veh-TAC and TUDCA-TAC groups** (refer to the legend to Figure H (C, D)). The heat map displays the number of upregulated (187, red) and downregulated (112, blue) genes. (B, C) The results are from the pathway enrichment analysis of Gene Ontology (GO) and Kyoto Encyclopedia of Genes and Genomes (KEGG) using the DEGs. Enrichment of GO and KEGG pathway was performed using DAVID bioinformatics resources 6.7 (<https://david.ncifcrf.gov/)>. Horizontal lines display cutoff for significance of (-log_10_ [*p*-value]) with Benjamini-Hochberg correction.


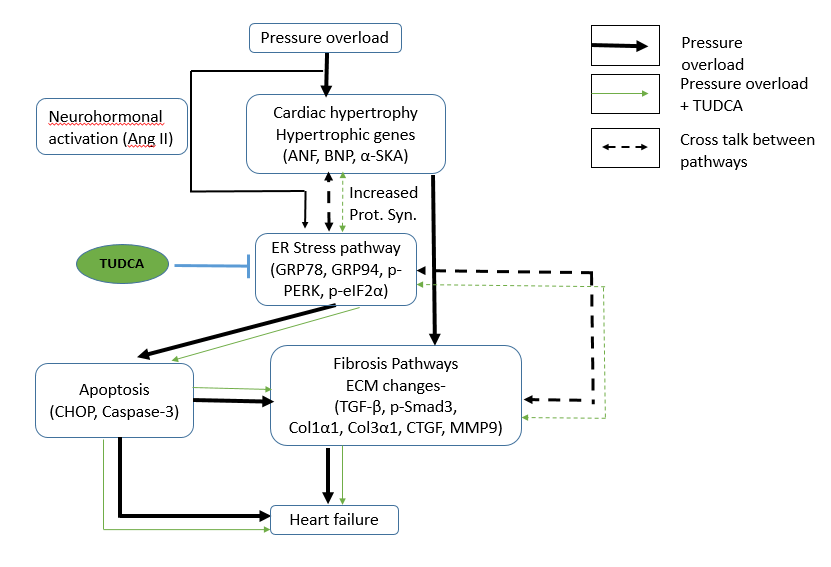


**Figure J. Schematic diagram depicting pressure overload–induced cellular events, which lead to intracellular ERS, and cardiac remodeling.** To the best of our knowledge, ERS and cardiac hypertrophy pathways cross talk in a reciprocal manner. Prolonged activation of ERS results in cardiac remodeling by inducing cardiomyocyte apoptosis, fibrosis, and eventually heart failure. TUDCA, as a chemical chaperone, inhibits ERS and there by protects the heart from pathological remodeling**.**
